# Supplementary material for: Evaluating knowledge fusion models on detecting adverse drug events in text
Source: PLOS Digit Health. 2025 Mar 18;4(3):e0000468. doi: 10.1371/journal.pdig.0000468 (PMC11918363; doi:10.1371/journal.pdig.0000468)
Supplement: S1 Note — Evaluation with GPT-based Model. (DOCX) [file pdig.0000468.s001.docx]

# S1 Note: Evaluation with GPT-based Model

The models utilized in our work are designed as token classifiers and were evaluated as such. The gpt-3.5-turbo model, referred to as GPT in this section, is however a generative model. Due to its generative characteristics, it could not directly be evaluated like the other models presented in this paper. Hence, we implemented two further approaches to evaluate GPT against the five corpora.

Firstly, GPT was instructed to generate a labeled sequence where each word of the input sequence was labeled as either being part of an ADR mention or not. This allowed us to evaluate GPT as if it was a token classification model. Secondly, GPT was asked to return the mentioned ADRs in a given input sequence as a list of spans.

The first approach was evaluated identically to the models in this work, by comparing the overlap of spans predicted by the model with the gold standard. However, this approach yielded poor F_1_-scores not surpassing 3% (CADEC: 0.71%, SMM4H: 2.20%, PsyTAR: 0.90%, ADE: 1.94%, TAC: 1.30%), and is therefore disregarded here. The results of the second approach are mentioned in the main manuscript (Results Section).

For both GPT evaluation strategies on the five corpora, a system prompt was constructed instructing the model to behave as expected. Furthermore, five random examples from the training set were added as context knowledge to further guide the model on the intended task. Neither approach benefited from adding more than five random examples to the system prompt.
