# Supplementary material for: Evaluating knowledge fusion models on detecting adverse drug events in text
Source: PLOS Digit Health. 2025 Mar 18;4(3):e0000468. doi: 10.1371/journal.pdig.0000468 (PMC11918363; doi:10.1371/journal.pdig.0000468)
Supplement: S1 Table — Final evaluation results on test set using link prediction as GNN training task. (DOCX) [file pdig.0000468.s002.docx]

# S1 Table: Final evaluation results on test set using link prediction as GNN training task

| **Model** | **Knowledge resource** | **Corpora** | | | | | | | | | | | | | | |
| --- | --- | --- | --- | --- | --- | --- | --- | --- | --- | --- | --- | --- | --- | --- | --- | --- |
|  |  | **CADEC** | | | **SMM4H** | | | **PsyTAR** | | | **ADE** | | | **TAC** | | |
|  |  | **P** | **R** | **F_1_** | **P** | **R** | **F_1_** | **P** | **R** | **F_1_** | **P** | **R** | **F_1_** | **P** | **R** | **F_1_** |
| Graph concat LP + BERT | DRUG | 66.86 | 75.46 | 70.90 | 58.57 | 72.03 | 64.60 | 63.55 | 81.23 | 71.31 | 80.86 | 79.02 | 79.93 | 91.97 | 94.48 | 93.21 |
| Graph concat LP + BERT | DRUGO_SYMP | 67.67 | 74.79 | 71.05 | 58.46 | 72.80 | 64.85 | 64.73 | 81.89 | 72.31 | 76.19 | 79.53 | 77.82 | 92.30 | 95.04 | 93.65 |
| Graph concat LP + BERT | SYMP | 68.84 | 75.04 | 71.81 | 59.05 | 71.26 | 64.58 | 63.70 | 83.60 | 72.30 | 76.01 | 80.62 | 78.25 | 92.47 | 95.44 | 93.93 |
| Graph conca LP + BioBERT | DRUG | 66.45 | 76.04 | 70.92 | 63.92 | 62.45 | 63.18 | 59.74 | 79.66 | 68.28 | 77.47 | 79.95 | 78.69 | 92.05 | 95.52 | 93.75 |
| Graph concat LP + BioBERT | DRUGO_SYMP | 67.19 | 74.87 | 70.83 | 57.09 | 64.75 | 60.68 | 63.71 | 81.10 | 71.36 | 78.93 | 77.00 | 77.95 | 92.21 | 94.72 | 93.44 |
| Graph concat LP + BioBERT | SYMP | 68.88 | 71.87 | 70.34 | 58.31 | 65.90 | 61.87 | 60.04 | 78.87 | 68.18 | 76.72 | 79.95 | 78.30 | 92.31 | 95.20 | 93.73 |
| Graph concat AW LP + BERT | DRUG | 64.79 | 75.88 | 69.90 | 57.64 | 69.35 | 62.96 | 64.21 | 84.78 | 73.08 | 76.74 | 79.78 | 78.23 | 91.73 | 94.16 | 92.93 |
| Graph concat AW LP + BERT | DRUGO_SYMP | 65.52 | 74.71 | 69.81 | 57.32 | 68.97 | 62.61 | 64.44 | 82.28 | 72.28 | 77.67 | 79.70 | 78.67 | 91.66 | 94.16 | 92.89 |
| Graph concat AW LP + BERT | SYMP | 63.06 | 76.13 | 69.30 | 58.31 | 71.26 | 64.14 | 66.08 | 83.33 | 73.71 | 78.78 | 80.37 | 79.57 | 92.94 | 94.80 | 93.86 |
| Graph concat AW LP + BioBERT | DRUG | 66.04 | 75.96 | 70.65 | 58.78 | 62.84 | 60.74 | 61.51 | 80.31 | 69.66 | 79.02 | 81.47 | 80.32 | 92.37 | 95.04 | 93.69 |
| Graph concat AW LP + BioBERT | DRUGO_SYMP | 66.67 | 75.29 | 70.72 | 57.63 | 65.13 | 61.15 | 65.27 | 77.43 | 70.83 | 74.56 | 81.21 | 77.74 | 92.13 | 94.72 | 93.41 |
| Graph concat AW LP + BioBERT | SYMP | 65.57 | 72.95 | 69.06 | 55.84 | 65.90 | 60.46 | 62.76 | 80.71 | 70.61 | 78.73 | 80.12 | 79.42 | 91.75 | 94.4 | 93.05 |

S1 Table: Final evaluation results on test set from graph concat model and graph concat model with adaptive GNN weights using link prediction as GNN training task. F_1_ stands for F_1_-score. All scores are strict scores and given in %. The best score on each corpus is given in bold. AW=adaptive weights. LP=link prediction.
