# Supplementary material for: Evaluating knowledge fusion models on detecting adverse drug events in text
Source: PLOS Digit Health. 2025 Mar 18;4(3):e0000468. doi: 10.1371/journal.pdig.0000468 (PMC11918363; doi:10.1371/journal.pdig.0000468)
Supplement: S2 Table — Results of hyperparameter tuning. (DOCX) [file pdig.0000468.s003.docx]

# S2 Table: Results of Hyperparameter Tuning

| Hyperparameter | Search Space | Models |
| --- | --- | --- |
| Number of layers in classification head | [1, 3] | Graph Concat (+AW) |
| Dropout probability | [0.2, 0.5] | BERT, BioBERT, Graph Concat (+AW), ERNIE |
| Learning rate | [8e-6, 1e-4] | BERT, BioBERT, Graph Concat (+AW), ERNIE |
| Batch size | [4,12] | BERT, BioBERT, Graph Concat (+AW), ERNIE |
| Weight decay | [4e-5, 1e-1] | BERT, BioBERT, Graph Concat (+AW), ERNIE |
| Learning rate of classification head | [1e-5, 1e-2] | Graph Concat (+AW), ERNIE |
| Learning rate of GNN | [1e-5, 1e-2] | Graph Concat AW |

S2 Table: Hyperparameter search space. The left column depicts the hyperparameters, the center column displays the search space, and the right column indicates for which model the hyperparameter was tuned. All models were tuned in 30 trials using Tree-structured Parzen Estimator (TPE) algorithm implemented in Optuna.
