# Supplementary material for: Evaluating knowledge fusion models on detecting adverse drug events in text
Source: PLOS Digit Health. 2025 Mar 18;4(3):e0000468. doi: 10.1371/journal.pdig.0000468 (PMC11918363; doi:10.1371/journal.pdig.0000468)
Supplement: S3 Table — Detailed evaluation results. (DOCX) [file pdig.0000468.s004.docx]

# S3 Table: Detailed Evaluation Results

| **Model** | **Knowledge resource** | **Corpora** | | | | | | | | | | | | | | |
| --- | --- | --- | --- | --- | --- | --- | --- | --- | --- | --- | --- | --- | --- | --- | --- | --- |
|  |  | **CADEC** | | | **SMM4H** | | | **PsyTAR** | | | **ADE** | | | **TAC** | | |
|  |  | **P** | **R** | **F_1_** | **P** | **R** | **F_1_** | **P** | **R** | **F_1_** | **P** | **R** | **F_1_** | **P** | **R** | **F_1_** |
| BERT | - | **70.29** | 73.46 | **71.84** | 59.38 | 65.52 | 62.30 | 64.36 | 76.77 | 70.02 | 75.34 | 75.4 | 75.37 | 90.42 | 93.76 | 92.06 |
| BioBERT | - | 67.29 | 74.71 | 70.81 | 57.57 | 67.05 | 61.95 | 60.85 | 79.13 | 68.80 | 77.63 | 81.3 | 79.42 | 92.8 | 94.96 | 93.87 |
| ERNIE + TransE | DRUG | 66.86 | **77.63** | **71.84** | 57.32 | 70.5 | 63.23 | 61.64 | 82.68 | 70.63 | 73.03 | 78.01 | 75.44 | 90.51 | 94.72 | 92.57 |
| ERNIE + TransE | DRUGO_SYMP | 65.62 | 73.46 | 69.32 | 59.36 | 64.37 | 61.76 | 62.13 | 82.68 | 70.95 | 74.86 | 77.25 | 76.04 | 90.7 | 94.48 | 92.55 |
| ERNIE + TransE | SYMP | 63.1 | 75.38 | 68.70 | 56.23 | 67.43 | 61.32 | **65.96** | 77.82 | 71.40 | 75.12 | 78.1 | 76.58 | 90.42 | 93.76 | 92.06 |
| Graph concat + BERT | DRUG | 67.53 | 73.62 | 70.45 | 58.22 | 67.82 | 62.65 | 64.74 | 79.53 | 71.38 | 78.17 | **81.47** | **79.79** | 92.59 | 95.04 | 93.8 |
| Graph concat + BERT | DRUGO_SYMP | 68.0 | 73.62 | 70.70 | 59.74 | 71.65 | **65.16** | 64.83 | 81.76 | 72.32 | 78.74 | 78.94 | 78.84 | 92.15 | 94.88 | 93.49 |
| Graph concat + BERT | SYMP | 68.36 | 73.96 | 71.05 | 57.69 | 68.97 | 62.83 | 63.74 | 82.81 | 72.03 | 76.02 | 80.37 | 78.13 | 92.8 | 94.96 | 93.87 |
| Graph concat + BioBERT | DRUG | 67.64 | 73.12 | 70.28 | 54.79 | 70.11 | 61.51 | 59.61 | 79.79 | 68.24 | 75.97 | 77.51 | 76.73 | 92.97 | **95.36** | **94.15** |
| Graph concat + BioBERT | DRUGO_SYMP | 65.68 | 73.96 | 69.57 | 58.67 | 67.43 | 62.75 | 63.69 | 77.82 | 70.05 | 79.04 | 78.77 | 78.90 | **93.14** | 94.64 | 93.88 |
| Graph concat + BioBERT | SYMP | 65.25 | 74.12 | 69.40 | 58.78 | 66.67 | 62.48 | 62.49 | 77.82 | 69.32 | 79.26 | 77.93 | 78.59 | 91.72 | 94.96 | 93.31 |
| Graph concat AW + BERT | DRUG | 66.37 | 75.29 | 70.55 | **61.79** | 66.28 | 63.96 | 63.93 | 83.73 | **72.50** | 78.54 | 79.53 | 79.03 | 91.61 | 94.48 | 93.02 |
| Graph concat AW + BERT | DRUGO_SYMP | 68.65 | 75.29 | 71.82 | 58.84 | 70.11 | 63.99 | 62.72 | 82.81 | 71.38 | **79.81** | 79.28 | 79.54 | 92.73 | 95.04 | 93.87 |
| Graph concat AW + BERT | SYMP | 67.87 | 73.54 | 70.59 | 58.97 | 70.5 | 64.22 | 64.36 | 81.76 | 72.02 | 77.62 | 79.19 | 78.4 | 91.84 | 94.64 | 93.22 |
| Graph concat AW + BioBERT | DRUG | 68.06 | 74.71 | 71.23 | 54.52 | 69.35 | 61.05 | 61.85 | 80.84 | 70.08 | 75.67 | 80.71 | 78.11 | 92.65 | 94.88 | 93.75 |
| Graph concat AW + BioBERT | DRUGO_SYMP | 65.01 | 73.21 | 68.87 | 58.45 | 66.28 | 62.12 | 61.45 | 80.31 | 69.62 | 78.39 | 78.85 | 78.62 | 92.79 | 94.8 | 93.78 |
| Graph concat AW + BioBERT | SYMP | 65.12 | 76.04 | 70.16 | 52.16 | 64.75 | 57.78 | 59.83 | 81.5 | 69.00 | 73.81 | 78.35 | 76.01 | 92.07 | 94.88 | 93.45 |
| Graph concat AWS + BERT | DRUG | 67.28 | 73.29 | 70.16 | 58.2 | **72.03** | 64.38 | 62.38 | **83.99** | 71.59 | 76.64 | 77.93 | 77.28 | 91.8 | 94.16 | 92.96 |
| Graph concat AWS + BERT | DRUGO_SYMP | 69.05 | 74.29 | 71.57 | 56.46 | **72.03** | 63.30 | 63.42 | 82.81 | 71.83 | 78.21 | 79.53 | 78.86 | 92.02 | 95.04 | 93.50 |
| Graph concat AWS + BERT | SYMP | 67.85 | 73.62 | 70.62 | 61.03 | 63.6 | 62.29 | 62.28 | 81.89 | 70.75 | 77.82 | 78.01 | 77.91 | 92.03 | 95.2 | 93.59 |
| Graph concat AWS + BioBERT | DRUG | 67.52 | 73.04 | 70.17 | 57.43 | 65.13 | 61.04 | 60.04 | 82.41 | 69.47 | 79.21 | 78.01 | 78.61 | 92.25 | 94.32 | 93.27 |
| Graph concat AWS + BioBERT | DRUGO_SYMP | 67.34 | 74.71 | 70.83 | 56.82 | 67.05 | 61.51 | 57.63 | 74.8 | 65.11 | 76.75 | 79.53 | 78.11 | 91.81 | 94.24 | 93.01 |
| Graph concat AWS + BioBERT + BioBERT | SYMP | 67.19 | 74.54 | 70.68 | 57.01 | 70.11 | 62.89 | 61.95 | 79.92 | 69.80 | 77.34 | 80.79 | 79.03 | 92.05 | 94.56 | 93.29 |

S3 Table: Final evaluation results on test set from all experiments including graph concat k-hop subgraph model. F_1_ stands for F_1_-score, P for Precision and R for Recall. All scores are strict scores and given in %. Best scores by corpus are given in bold.
